# The infected diabetic foot: Incidence and risk factors for dehiscence after surgery for diabetic foot infections

**DOI:** 10.1111/wrr.13235

**Published:** 2024-12-15

**Authors:** Lawrence A. Lavery, Mario C. Reyes, Bijan Najafi, Tyler L. Coye, Jayer Chung, Michael C. Siah, Arthur N. Tarricone

**Affiliations:** ^1^ Department of Plastic Surgery University of Texas Southwestern Medical Center Dallas Texas USA; ^2^ Department of Surgery Baylor College of Medicine Houston Texas USA; ^3^ Department of Surgery University of Texas Southwestern Medical Center Dallas Texas USA

**Keywords:** amputation, diabetes, infection, osteomyelitis, ulcer

## Abstract

Our objective was to assess the incidence, risk factors and clinical outcomes of dehiscence after foot surgery in diabetic patients. We used pooled patient‐level data from two randomised clinical trials with 240 diabetic patients who required foot surgery for infections. Most patients (*n* = 180, 75.0%) had surgical wound closure. We defined dehisced surgical wounds (DSW) when the surgical site was not completely epithelialized with no drainage after sutures/staples were removed with a 2‐week validation of healing. We evaluated the time to heal, re‐infection, re‐ulceration, hospital admissions and amputations. Moderate and severe infection was based on criteria of the International Working Group on the Diabetic Foot. We used *χ* ^2^ and *t*‐test and Mann–Whitney *U* for comparison of clinical events, with *α* of <0.05. DSW occurred in 137 (76.1%) patients. DSW patients were more likely to have hypertension (62.8% vs. 81.8%, *p* = 0.01), high ESR (59.1 ± 37.9 vs. 75.9 ± 37.6, *p* = 0.01), low toe brachial indices (0.8 ± 0.2) (0.7 ± 0.2, *p* = 0.005), toe brachial indices <0.6 (16.7% vs. 40.9%, *p* = 0.008), and low skin perfusion pressure measurements (dorsal medial 71.0 ± 29.4 vs. 59.3 ± 23.3, *p* = 0.01, and plantar medial 81.8 ± 24.9 vs. 72.2 ± 20.4, *p* = 0.02). During 12‐month follow‐up, DSW patients were 12.9 times more likely to have re‐infection (0% vs. 12.4%, *p* = 0.02) and 6.8 times more likely to require amputation (2.3% vs. 13.9%, *p* = 0.04). The median healing time (28, 22.5–35.0 vs. 114.0, 69.0; 365, *p* = 0.001), and median length of hospitalisation were longer in DSW patients (12.0, 9.01–9.0 vs. 15.0, 11.0–24.0, *p* = 0.04). There was a high incidence of DSW, associated with poor clinical outcomes.

**Abbreviations**
BMI: body mass index
CHF: congestive heart failure
CI: confidence Interval
CKD: chronic kidney disease
CRP: C‐reactive protein
CT: computerized tomography
eGFR: estimated glomerular filtration rate
ESRD: end stage renal disease
HIV: human immunodeficiency virus
IDSA: Infectious Disease Society of America
IQR: interquartile range
MI: byocardial infarction
MRI: magnetic resonance imaging
SIRS: Systemic Inflammatory Response Syndrome
SPEC CT: single‐photon emission computerized tomography
SPP: skin perfusion pressure
VPT: vibration perception threshold
WBC: white blood cell count

## 1 INTRODUCTION

After surgical closure of a lower extremity amputation, wound failure is common. There are few reports concerning the incidence of surgical site wound failure after amputation in people with diabetes. Brown and Wukich reported that 24%–30% of patients with diabetes that required a below‐the‐knee amputation had wound dehisce and infection and required surgical revision.^1^ Wound failure is more common in foot amputations, probably because there is more small vessel disease and the foot is exposed to repetitive trauma from walking or transferring on the amputated foot. Below the knee and above the knee amputations are usually not exposed to repetitive injury during the immediate postoperative period. After surgical closure of a foot amputation or foot wound, the incidence of wound dehiscence in patients with diabetes ranges from 40% to 61%.^2^, ^3^, ^4^, ^5^ When there is wound dehiscence, patients are essentially exposed to the same cycle of prolonged healing, infection, hospitalisation and amputation that they experienced with their initial foot ulceration. Unfortunately, we have not been able to identify any studies that identify risk factors and long‐term clinical outcomes after surgical wound dehisces. This paper's objectives are to identify the incidence of wound dehiscence, the risk factors for wound dehiscence, and the clinical outcomes after surgical wound dehiscence.

## 2 METHODS

### 2.1 Study design and participants

This is a post hoc analysis using pooled patient‐level data from two randomised controlled trials^2^, ^3^ conducted by our group. These trials employed uniform evaluation criteria and definitions for outcomes and adverse events. We included 240 patients aged 18–89 years with moderate to severe foot infections, as defined by the International Working Group on the Diabetic Foot (IWGDF) guidelines.^6^ We used either positive bone culture or bone histopathology to confirm the diagnosis of osteomyelitis. A negative MRI, SPECT CT or bone biopsy were used to rule out bone infection. Only diabetic patients that had closure of a foot surgical wound with a 12‐month follow‐up were included. During their index hospitalisation, 180 patients underwent surgery for infection and had their wounds surgically closed.

### 2.2 Clinical measurements

Peripheral sensory neuropathy was identified using either an abnormal vibration perception threshold (VPT using Salix Medical devices, San Antonio, TX, USA) or the 10‐g Semmes‐Weinstein monofilament test. Vascular assessments included toe systolic pressures and brachial indices, obtained from arterial Doppler studies. We stratified ankle‐arm systolic blood pressure ratio (ABI) into three categories: less than 0.90, 0.9–1.30, and greater than 1.30.^7^ Skin perfusion pressures on the dorsum and sole of the foot were measured using Sensilase technology (Vasamed, Eden Prairie, MN, USA). Infections were classified based on IWGDF/IDSA criteria, with osteomyelitis confirmed either by positive bone culture or intraoperative pathology.^6^, ^8^

### 2.3 Surgical and postoperative procedures

The standard treatment for diabetic foot infections required initiating empiric antibiotic therapy, followed by collection of deep tissue and bone samples for culture and histological examination during surgery. Patients typically returned to the operating room within 48–72 h for additional interventions, such as repeat incision and drainage, amputation or late wound closure, depending on surgical assessment. Wound closure techniques varied according to surgeon preference, and postoperative care included the use of casts, removable boots, or healing sandals to off‐load pressure from the affected area, tailored to each patient's wound location and stability. A healed surgical wound was defined as having no drainage and complete epithelialization at the time that sutures were removed, typically 2–3 weeks post‐surgery. This was then verified after 2 weeks to determine that surgical site was healed. A dehisced surgical wound (DSW) was characterised by ongoing drainage or incomplete re‐epithelialization post‐suture removal.

### 2.4 Statistical analysis

We described categorical variables as frequencies and percentages, and continuous variables as means and standard deviations. Differences between patients with and without wound dehiscence were analysed using the chi‐squared test of homogeneity or Fisher exact test for categorical variables and the Mann–Whitney *U* test for continuous variables. Relative risk assessments were conducted to explore the correlation between wound dehiscence, risk factors and clinical outcomes.

## 3 RESULTS

Among the 180 patients with surgical wound closure, 137 (76.1%) had wound dehiscence of all or part of the surgical site. There were few differences in the baseline characteristics of people that healed their surgical site and patients that experienced a DSW (Table 1). Patients with DSWs were significantly more likely to have hypertension (62.8% vs. 81.8%, *p* = 0.01), high ESR (59.1 ± 37.9 vs. 75.9 ± 37.6, *p* = 0.01), low toe brachial indices (0.8 ± 0.2 vs. 0.7 ± 0.2, *p* = 0.005), toe brachial indices <0.6 (16.7% vs. 40.9%, *p* = 0.008), and low skin perfusion pressure measurements (dorsal medial 71.0 ± 29.4 vs. 59.3 ± 23.3, *p* = 0.01), and plantar medial (81.8 ± 24.9 vs. 72.2 ± 20.4, *p* = 0.02). In addition, during the index hospitalisation there was no difference in the incidence of amputations (65.1% vs. 65.0%); however, DSW patients required significantly longer median length of hospitalisation (11.0, 8.0–12.5 vs. 12.0, 9.0–16.0, *p* = 0.01).

**TABLE 1:** Risk factors in patients with and without surgical site dehiscence after diabetic foot infection surgery.

	No dehiscence (*n* = 43)	Dehiscence (*n* = 137)	(95% CI)	*p*‐Value
Age	51.1 (9.9)	50.6 (9.5)	−3.8 to 2.8	0.78
Male	29 (67.4)	109 (79.6)	1.8 (0.8–4.0)	0.10
BMI (kg/m^2^)	31.2 (6.9)	31.6 (7.9)	−2.2 to 3.2	0.74
Race				
Non‐Hispanic White	11 (25.6)	32 (23.4)	0.9 (0.4–2.0)	0.77
African descent	10 (23.3)	45 (32.8)	1.6 (0.7–3.6)	0.23
Hispanic	22 (51.2)	60 (43.8)	0.7 (0.4–1.5)	0.40
Social factors				
<12 Years of education	23 (53.5)	75 (57.7)	1.1 (0.5–2.3)	0.78
Spanish language	9 (20.9)	25 (18.2)	0.8 (0.4–1.9)	0.82
Living alone	4 (9.3)	10 (7.3)	0.8 (0.2–2.6)	0.74
Household ambulatory	4 (9.3)	3 (2.2)	0.2 (0.0–1.0)	0.06
Married	13 (30.2)	44 (32.1)	1.1 (0.5–2.3)	0.85
Tobacco use	9 (20.9)	29 (21.2)	1.0 (0.4–2.4)	0.97
Alcohol use	12 (27.9)	32 (23.4)	0.8 (0.4–1.7)	0.55
Illicit drug use	1 (2.3)	10 (7.3)	3.3 (0.4–26.6)	0.24
Medical history				
Hypertension	27 (62.8)	112 (81.8)	2.6 (1.2–5.7)	**0.01**
MI	4 (9.3)	9 (6.6)	0.7 (0.2–2.4)	0.55
CHF	6 (14.0)	13 (9.5)	0.6 (0.2–1.8)	0.41
HIV	0 (0.0)	2 (1.5)	1.6 (0.1–34.3)	0.42
Retinopathy	8 (18.6)	28 (20.4)	1.1 (0.5–2.7)	0.79
CKD I–IV	9 (20.9)	37 (27.0)	1.4 (0.6–3.2)	0.43
ESRD	4 (9.3)	8 (5.8)	0.6 (0.2–2.1)	0.43
Sensory neuropathy	42 (97.7)	131 (96.3)	0.6 (0.1–5.5)	0.67
Abnormal monofilament	39 (92.9)	115 (86.5)	0.5 (0.1–1.8)	0.27
VPT forefoot	45.6 (17.1)	47.6 (23.5)	−5.6 to 9.7	0.60
Charcot arthropathy history	0 (0.0)	11 (8.0)	7.9 (0.5–137.0)	0.06
Prior amputation	21 (48.8)	65 (47.4)	0.9 (0.5–1.8)	0.87
Medications				
Insulin	31 (72.1)	92 (67.2)	0.8 (0.4–1.7)	0.54
Calcium channel blockers	11 (25.6)	35 (25.5)	1.0 (0.5–2.2)	0.99
Steroids	3 (7.0)	9 (6.6)	1.1 (0.3–4.1)	0.93
Beta‐blockers	12 (27.9)	36 (26.3)	0.9 (0.4–2.0)	0.83
Gabapentin	9 (20.9)	48 (35.0)	2.0 (0.9–4.6)	0.08
Pre‐gabalin	2 (4.7)	4 (2.9)	0.6 (0.1–3.5)	0.58
Admission characteristics				
Osteomyelitis	33 (76.7)	102 (74.5)	0.8 (0.4–2.0)	0.76
SIRS criteria	4 (9.3)	25 (18.2)	2.2 (0.7–6.7)	0.16
Temperature >38.6	3 (7.0)	20 (14.3)	2.3 (0.6–8.1)	0.19
Heart rate >90	13 (30.2)	49 (35.8)	1.3 (0.6–2.7)	0.51
Respiratory rate >20	6 (14.0)	12 (8.9)	0.6 (0.2–1.7)	0.34
WBC >12,000	10 (23.3)	36 (26.3)	1.2 (0.5–2.6)	0.69
Admissions labs				
CRP	10.2 (14.3)	9.9 (7.4)	−4.1 to 3.5	0.88
ESR	59.1 (37.9)	75.9 (37.6)	3.4–30.1	**0.01**
Glycated haemoglobin (%)	9.5 (2.6)	9.9 (3.6)	−0.7 to 1.7	0.43
eGFR	51.6 (17.9)	51.2 (15.7)	−6.1 to 5.2	0.87
Wound characteristics				
Ulcer duration	69.5 (96.8)	104.7 (226.8)	−35.2 to 105.5	0.32
Median (IQR)	30.0 (15.0–60.0)	30.0 (10.0–60.0)		0.92
Wound area (cm^2^)	12.3 (13.6)	14.9 (14.8)	−2.4 to 7.6	0.64
Median (IQR)	7.2 (3.9–16.3)	9.7 (5.6–16.1)		0.06
Wound volume (cm^3^)	14.6 (33.9)	17.5 (34.5)	−9.1 to 14.6	0.31
Median (IQR)	2.9 (1.0–13.4)	7.2 (2.4–14.4)		0.08
Ankle brachial index				
<0.90	3 (7.0)	12 (8.8)	1.3 (0.3–4.8)	0.70
0.90–1.30	27 (62.8)	75 (55.1)	0.7 (0.4–1.5)	0.38
>1.30	13 (30.2)	49 (36.0)	1.3 (0.6–2.7)	0.49
Toe brachial index (*n* = 146)	0.8 (0.2)	0.7 (0.2)	−0.2 to 0.0	**0.005**
TBI <0.6	6 (16.7)	45 (40.9)	3.5 (1.3–9.0)	**0.008**
Monckeberg's sclerosis	24 (55.8)	78 (56.9)	1.1 (0.5–2.1)	0.88
SPP dorsal medial	71.0 (29.4)	59.3 (23.3)	−20.6 to 2.7	**0.01**
SPP dorsal medial ≤ 40 mmHg	5 (13.3)	34 (26.2)	2.4 (0.9–6.7)	0.08
SPP dorsal lateral	70.2 (26.9)	62.8 (24.1)	−16.3 to 1.5	0.11
SPP dorsal lateral ≤40 mmHg	3 (7.7)	26 (20.0)	2.9 (0.8–10.4)	0.08
SPP plantar medial	81.8 (24.9)	72.2 (20.4)	−17.3 to 1.8	**0.02**
SPP plantar medial ≤40 mmHg	2 (5.1)	8 (6.1)	1.2 (0.2–5.9)	0.83
SPP plantar lateral	81.5 (18.6)	75.9 (24.1)	−13.9 to 2.9	0.20
SPP plantar lateral ≤40 mmHg	0 (0.0)	13 (10.0)	8.5 (0.5–147.0)	0.05

*Note*: Bold values are statistically significant.

**FIGURE 1:**
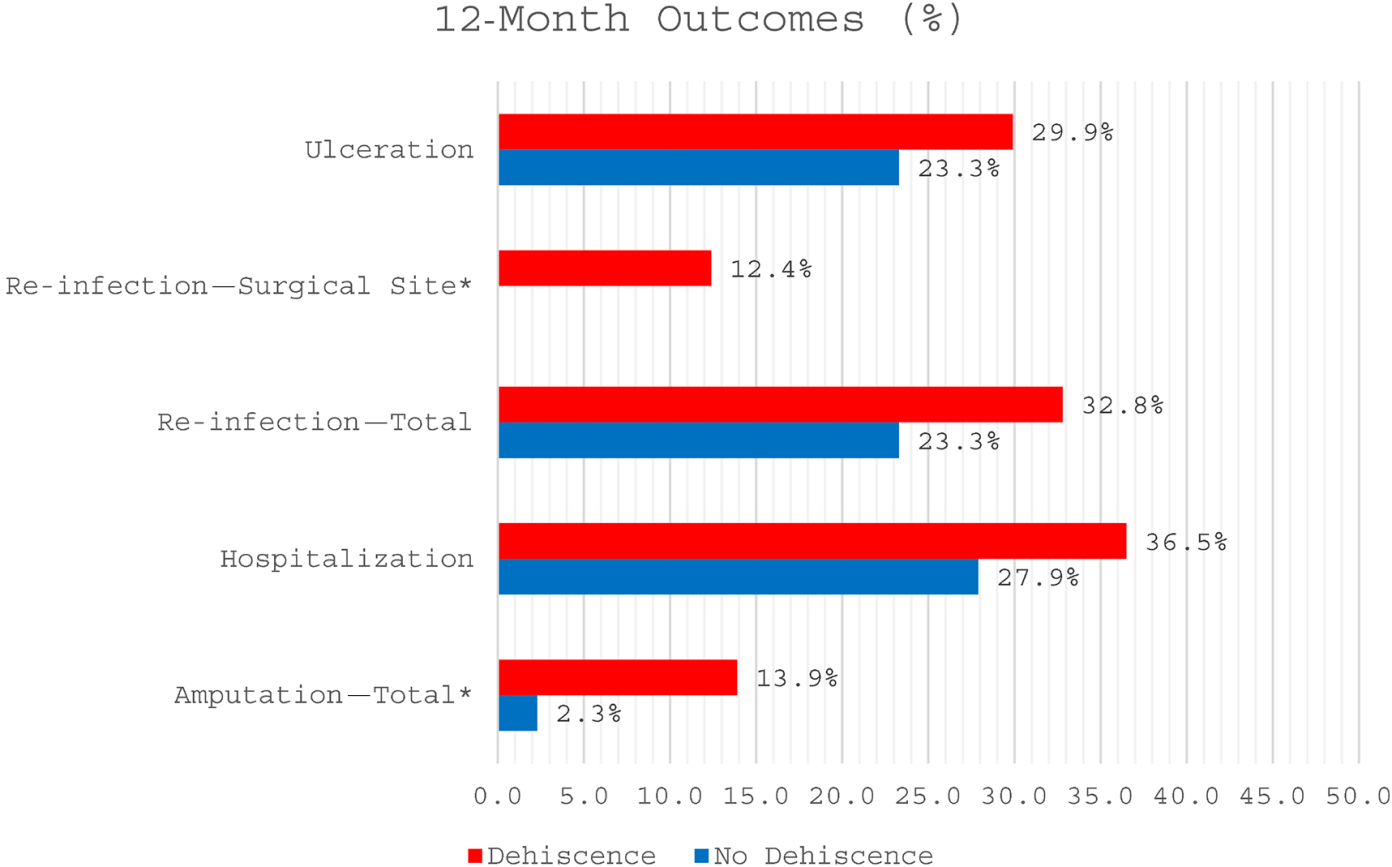
Twelve‐month outcomes comparison of patients with and without surgical site dehiscence after diabetic foot infection surgery. This bar chart compares the 12‐month clinical outcomes in patients with and without dehisced surgical wounds after diabetic foot infection surgery. Patients with dehiscence were 12.9 times more likely to have re‐infection at the surgical site (0% vs. 12.4%, *p* = 0.02). Dehisced surgical wounds were 6.8× more likely to require amputation (2.3% vs. 13.9%, *p* = 0.04). “*” is statistically significant, matching the bold values in Table 2.

During the 12‐month follow‐up, patients with DSWs had more complications compared to patients that healed their index surgical site (Table 2). Patients with DSWs were 12.9 times more likely to have re‐infection at the surgical site (0% vs. 12.4%, *p* = 0.02) and 6.8 times more likely to require amputation (2.3% vs. 13.9%, *p* = 0.04). As expected, the median time to heal was significantly longer in people with DSWs (28, 22.5–35.0 vs. 114.0, 69.0;365, *p* = 0.001), and they had significantly longer median length of hospitalisation (12.0, 9.01–9.0 vs. 15.0, 11.0–24.0, *p* = 0.04). There were no differences in ulceration (23.3% vs. 29.9%, *p* = 0.40), all‐cause hospitalizations (27.9% vs. 36.5%, *p* = 0.30), foot‐related hospitalizations (9.3% vs. 21.9%, *p* = 0.06), and median days of antibiotic treatment (42.0, 21.5–52.0 vs. 45.0, 23.0–71.0, *p* = 0.21) (Figure 1).

**TABLE 2:** Clinical outcomes in patients with and without surgical site dehiscence after diabetic foot infection surgery.

	No dehiscence (*n* = 43)	Dehiscence (*n* = 137)	(CI 95%)	*p*‐Value
Index hospital admission				
Amputation	28 (65.1)	89 (65.0)	1.0 (0.5–2.0)	0.99
Length of stay (days) Median (IQR)	11.0 (8.0–12.5)	12.0 (9.0–16.0)		**0.01**
Antibiotic days median (IQR)	21.0 (13.5–42.0)	21.0 (17.0–44.0)		0.50
12‐Month follow‐up				
Time to heal (days) *Median* (*IQR*)	28.0 (22.5–35.0)	114.0 (69.0–365)		**0.001**
Re‐infection—all sites	10 (23.3)	45 (32.8)	1.6 (0.7–3.6)	0.23
Re‐infection—surgical site	0 (0.0)	17 (12.4)	12.9 (0.7–234.0)	**0.02**
Ulceration	10 (23.3)	41 (29.9)	1.4 (0.6–3.1)	0.40
Admission—all cause	12 (27.9)	50 (36.5)	1.5 (0.7–3.2)	0.30
Admission—foot	4 (9.3)	30 (21.9)	3.3 (0.8–12.6)	0.06
Amputation	1 (2.3)	19 (13.9)	6.8 (0.9–52.1)	**0.04**
Foot amputation	1 (2.2)	12 (8.6)	0.56 (0.0–15.5)	0.45
Leg amputation	0 (0.0)	7 (5.1)	1.8 (0.1–50.1)	0.45
Total length of stay (days) Median (IQR)	12.0 (9.0–19.0)	15.0 (11.0–24.0)		**0.04**
Total antibiotic days Median (IQR)	42.0 (21.5–52.0)	45.0 (23.0–71.0)		0.21

*Note*: Bold values are statistically significant.

## 4 DISCUSSION

The results of this study show the high incidence of failed surgical wound closure in patients with diabetic foot infections and the clinical outcomes that result after wound failure. We have not been able to identify any studies that evaluate risk factors for wound failure. The risk factors we identified included markers for peripheral arterial disease (low toe brachial pressures, low skin perfusion pressure), as well as hypertension, and erythrocyte sedimentation rate. These factors have been associated with poor wound healing. In contrast, there was no difference in the history of amputation, insulin, steroids, glucose control, albumin, osteomyelitis and infection severity in patients with healed surgical wounds and failed surgical wound closure.

This is not a topic that is often addressed in detail in diabetic foot ulcer and amputation publications. The reported incidence of wound failure is often very high^1^, ^5^, ^9^; however, we have been unable to identify studies that provide a clear operational definition of a DSW, measured the proportion of the wounds that failed, or measured the size of the dehisced wound. Sometimes dehiscence, infection, and necrosis are grouped under the umbrella of ‘stump or wound complications,’ so it is difficult to understand the nuances of this problem. We have not found any publications that specifically report the clinical outcomes associated with dehisced surgical foot wounds. We identified an increase in complications such as re‐infection, hospitalisation and amputation due to wound dehiscence. Re‐infection and amputation were 12.9 and 6.8 times higher in people with failed surgical wounds. Interestingly, the incidence of infection at other sites was still 23.3% in people that healed their index surgery, because these patients developed new ulcers during the follow‐up period.

There are limitations and advantages in the methodology of this study. This study was from one group at two hospitals. Generalizability of our data would have been better if we included multiple sites. One of the shortcomings of this paper was that we did not measure the size of DSWs. We counted any failure of the surgical wound as a dehiscence, but we did not record area, volume, or depth. In addition, this was a selective patient population. Study subjects were admitted to the hospital with foot infections that required surgery. We did not evaluate the types of closures (sutures or staples), the experience of the surgeon, postoperative off‐loading or patient activity, which could have contributed to wound failure.

The main advantage of this study was that variables such as medical history, initial wound measurements, vascular studies and social determinants of health were collected prospectively. One of the unmet needs in people with diabetic foot wounds and infections is assessment of perfusion. This population has a very high rate of Monckeberg's sclerosis and non‐compressible arteries,^10^, ^11^ which makes many of these studies unreliable. Toe brachial indices and skin perfusion pressure measurements have been reported to be good studies to predict healing, and they were effective in this study.^12^ Unfortunately, many high‐risk people have a history of toe amputation. We were only able to use TBIs in 146 (81.1%) of the subjects in the study.

## 5 CONCLUSION

Dehiscence after surgical closure in diabetic patients that were admitted to hospital for infection is very common. More importantly, dehisced surgical foot wounds lead to a cascade of complications, including infections, hospital admission and amputation. Understanding the risk factors for wound failure is one of the first steps in reducing dehiscence. Future work in this area should provide wound area and volume measurements and evaluate the factors we did not include in this study such as they type of off‐loading and specific types of sutures and closure techniques.

## AUTHOR CONTRIBUTIONS

We would like to thank each author's contribution to the manuscript. The study design was developed by Dr. Lawrence A. Lavery. Dr. Mario C. Reyes performed data collection and extraction, statistical analysis and manuscript review. Dr. Arthur N. Tarricone performed statistical analysis and manuscript review. Dr. Tyler L. Coye, Dr. Bijan Najafi, Dr. Jayer Chung, Dr. Michael C. Siah, and Dr. Lawrence A. Lavery performed manuscript writing, review and editing.

## CONFLICT OF INTEREST STATEMENT

None of the authors have any conflicts of interest that pertain to this research.

## Data Availability

The data that support the findings of this study are available from the corresponding author, upon reasonable request.
